# Hypoxemia and Postoperative Monitoring After Anesthesia: A Prospective Observational Study Using Portable Pulse Oximetry in a Resource-Limited Setting in Guatemala

**DOI:** 10.7759/cureus.78075

**Published:** 2025-01-27

**Authors:** Ryan A Turner, Colby G Simmons, Sindy Ramirez, Jakob E Gamboa

**Affiliations:** 1 Anesthesiology, University of Colorado School of Medicine, Aurora, USA; 2 Anesthesiology, Hospital Nacional de Coatepeque, Coatepeque, GTM

**Keywords:** global health, patient safety, postoperative monitoring, pulse oximetry, resource-limited setting

## Abstract

Pulse oximetry is a critical component of patient monitoring to ensure adequate oxygenation in the perioperative period. However, its use remains limited in low- and middle-income countries due to device scarcity, limited funding, and lack of training. This prospective observational study describes the incidence of early postoperative hypoxemia (EPH) with newly implemented portable pulse oximetry and associated factors that impact postoperative management at the Hospital Nacional de Coatepeque (HNC), a primary referral public hospital in Guatemala. Semi-structured interviews were conducted with perioperative medical staff to explore perspectives regarding postoperative monitoring and patient safety in a resource-limited setting. One hundred patients were included, of which 10% experienced EPH. Patient age was significantly associated with EPH. The average duration in the recovery area of 14 minutes, with a lack of subsequent monitoring, was a primary concern of the 14 interviewed medical personnel. The greatest perceived needs include enhanced monitoring, increased staffing, and a dedicated post-anesthesia care unit. Pulse oximetry is essential to detect previously unrecognized EPH. Improved postoperative monitoring and increased recovery time and staffing are priorities to enhance patient safety at public hospitals in Guatemala.

## Introduction

Monitoring tissue oxygen saturation through pulse oximetry has become an international standard of care, providing valuable information about a patient’s clinical status to ensure adequate oxygenation in the perioperative period [1]. Yet, pulse oximetry remains underutilized in some areas in low- and middle-income countries (LMICs) due to barriers such as device scarcity, insufficient funding, and lack of training. Research has demonstrated that the implementation of portable pulse oximetry is a cost-effective intervention in resource-limited settings that enables early detection of hypoxia before irreversible damage occurs [2-5].

In recent years, large-scale initiatives have expanded the availability and broader use of pulse oximetry in many LMICs, leading to significant improvements in perioperative care and patient safety [2,3,6]. However, most of these initiatives have taken place in Africa and Southeast Asia. There are no documented studies of implementing portable pulse oximetry in first-referral hospitals in Guatemala. Due to limited capacity and infrastructure, data on complications arising from a lack of monitoring is scarce and not widely available.

In Guatemala, 88% of health care is provided through the public health system. Limitations in funding, equipment, and workforce can impact care delivery in public hospitals, especially in more remote regions of the country. For example, one survey of national capacity reported that only 70% of district hospitals had a consistent supply of oxygen, and only 50% of national hospitals reported always or almost always having pulse oximeters [7]. In southwestern Guatemala, the Hospital Nacional de Coatepeque (HNC) is a first-referral, public hospital that provides care to high-risk surrounding rural communities in Coatepeque, Quetzaltenango District, Guatemala [8]. We performed a needs assessment at the HNC in 2023 which revealed a lack of postoperative physiologic monitoring and a designated post-anesthesia care unit (PACU). After surgery, patients at HNC wait in a holding area until nursing staff become available to transfer them to their respective medical units.

Through the Safe Surgery Initiative of the non-profit AmeriCares organization, two portable pulse oximeters (CMI Health Handheld Pulse Oximeter PC66-H, CMI Health, Alpharetta, GA) were provided as donations to the hospital for monitoring patients during the immediate post-surgical period. These devices are rechargeable and HNC has reliable access to power for recharging. Utilizing newly implemented pulse oximetry, we investigate patient and health system factors that impact postoperative patient care and outcomes in a resource-limited hospital in Guatemala. Through pulse oximetry implementation, we expect an increased detection of hypoxemic events and the associated risk factors. Additionally, we explore the perceptions of hospital staff concerning the health system and process factors that impact postoperative care and safety.

## Materials and methods

This prospective observational study describes the incidence of early postoperative hypoxemia (EPH) and the management of patients in the immediate post-surgical setting at the HNC. Hypoxemic events, defined as periods with oxygen saturation (SpO_2_) less than 90%, were previously undetectable due to a lack of essential resources, including a designated PACU and pulse oximeter monitoring. This study was approved by the Colorado Multiple Institutional Review Board and local K’Awil ethics committee and adheres to the applicable Enhancing the QUAlity and Transparency Of health Research (EQUATOR) Network guidelines for The Strengthening the Reporting of Observational Studies in Epidemiology (STROBE) [9].

Data regarding the early postoperative course and oxygen saturation levels were collected from a convenience sample of patients who presented after surgery at the HNC during four one-week periods from May to August 2024. The consent process was conducted in Spanish by language-certified research team members or the local care team, and informed consent was obtained from patients preoperatively. Patients were notified of their right to refuse participation without any consequences to their medical care. All patients were provided the opportunity to ask questions during the initial consent and at any point to ensure full understanding. In illiterate patients who were unable to sign, hospital protocols were followed with fingerprint stamps, and consent materials were verbally presented to patients. Exclusion criteria included inability to consent, refusal, or age less than one year. All other patients who consented to participate were enrolled in the study. Research team members who were not directly involved in the perioperative care observed the patients in the postoperative holding area and recorded the occurrence and characteristics of EPH, postoperative management, surgery and anesthesia type, and basic patient information (age, gender, body mass index [BMI], American Society of Anesthesiologists [ASA] score). Information was stored without direct identifiers in a secure online database that was only accessible to the research team.

Additionally, semi-structured interviews were performed in Spanish with perioperative HNC staff who consented to participate. These interviews examined the providers’ perceptions of patient safety needs and concerns in the perioperative setting. Due to a lack of existing validated surveys that addressed our study objectives, we designed a brief interview guide that consisted of two primary questions: 1. What are the greatest perioperative needs in the hospital? 2. What are your greatest concerns for patient safety? After prompting the questions, interviewees were given the opportunity to freely discuss any additional topics. All interviews lasted between 2-8 minutes and were audio-recorded using a secure, encrypted mobile application. Participants were assured that responses would not affect employment, and only their profession was recorded.

Descriptive statistics were calculated and presented as frequencies with percentages, means, and standard deviations (SD). A multiple logistic regression was used to test for a relationship of experiencing a hypoxemic event with relevant clinical factors using Stata statistical software (StataCorp LLC, College Station, TX, 2023). A Bonferroni correction was performed to reduce the risk with multiple testing, and a modified statistical significance level of p <0.01 was considered significant. The sample size was based on the number of patients available during the study period, due to the severe local staffing constraints and the availability of research team support. Interview participants were selected from each relevant specialty, and we aimed to recruit at least 12 individuals to reach saturation of relevant themes. Audio recordings were reviewed, and primary codes were developed for each interview by two research team members. Coded data was analyzed for major themes between profession types and then counted and displayed as frequencies among respondents.

## Results

A total of 100 patients were included, 41% of whom were female (Table 1). Ten (10%) patients had at least one recorded episode of hypoxemia during the immediate postoperative period, with the lowest recorded oxygen saturation of 86% (Table 2). The longest hypoxic event lasted 90 seconds, with stimulation as the primary intervention. Only one patient during the study received supplemental oxygen therapy in the postoperative area.

**Table 1:** Patient factors stratified by occurrence of hypoxemic events. EPH, early postoperative hypoxemia; SD, standard deviation; BMI, body mass index; ASA, American Society of Anesthesiologists; OB/GYN, Obstetrics and Gynecology. *A p-value <0.01 was considered statistically significant after Bonferroni correction.

	Patients without EPH (n=90)	Patients with EPH (n=10)	Total (n=100)	p-Value*
Age				
Mean (SD)	37.7 (19.5)	61.4 (19.1)	40.2 (20.7)	0.004
Sex				
No of females	35	5	40	0.515
BMI				
Mean (SD)	24.5 (4.6)	28.6 (5.8)	25.0 (4.9)	0.048
ASA score				
Mean (SD)	1.7 (0.7)	2.4 (0.7)	1.8 (0.7)	0.611
Type of anesthesia, n (%)				
General	8 (9%)	2 (20%)	10 (10%)	0.013
Spinal	44 (49%)	5 (50%)	49 (49%)	-
Peripheral nerve block	52 (58%)	3 (50%)	55 (55%)	-
Type of surgery, n (%)				
Orthopedic/Trauma	62 (69%)	6 (50%)	68 (68%)	-
General	12 (13%)	2 (30%)	14 (14%)	-
OB/GYN	16 (18%)	2 (20%)	18 (18%)	-

**Table 2:** Characteristics of hypoxemic events. SpO_2_, oxygen saturation; ICU, intensive care unit.

Number of episodes	10
Lowest recorded SpO_2_	86%
Average duration of hypoxemia	15 seconds
Intervention	
Verbal stimulation	1
Tactile stimulation	3
Oxygen	0
None	6
Supplemental oxygen	
Yes	0
No	10
Postoperative analgesics	
Yes	0
No	10
Disposition	
Floor	10
ICU	0

Age was significantly associated with EPH (p=0.004), with an odds ratio of 1.11 (99% confidence interval: 1.03431, 1.193091). The average duration in the immediate postoperative area before transfer to the floor was 14 (SD 8.6) minutes. Of note, there was no staff present during the entirety of the recovery period to monitor patients while awaiting transfer to their respective hospital units.

**Figure 1:**
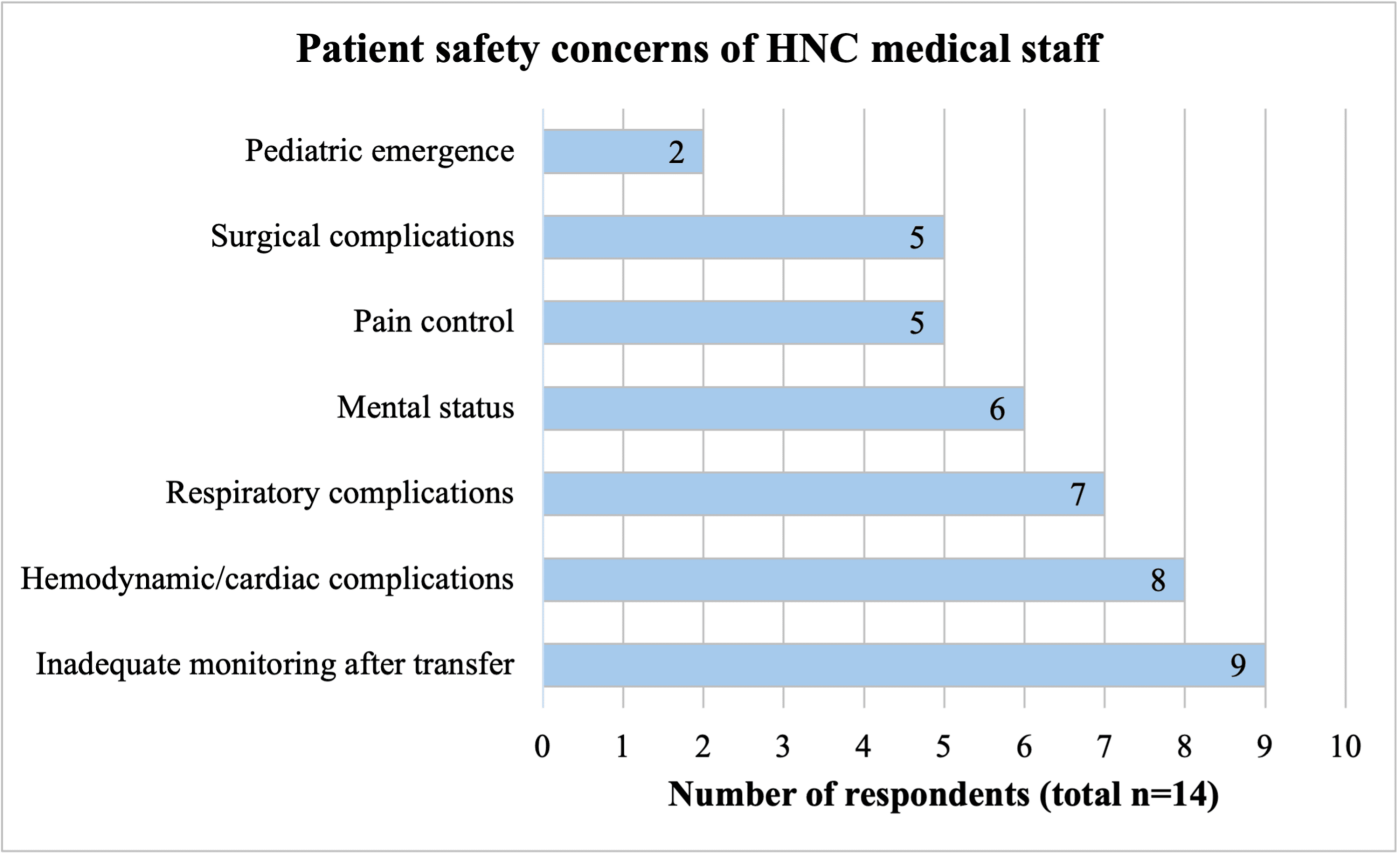
Primary patient safety concerns expressed by HNC medical staff, by number of respondents. HNC, Hospital Nacional de Coatepeque.

**Figure 2:**
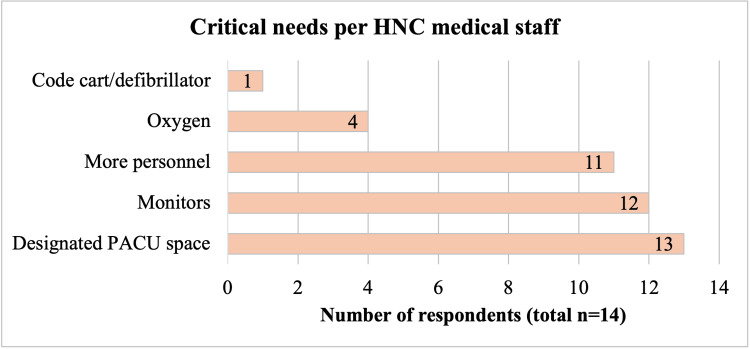
Critical needs for postoperative patient safety identified by HNC medical staff, by number of respondents. HNC, Hospital Nacional de Coatepeque; PACU, post-anesthesia care unit.

Interviews were performed with 14 HNC medical personnel, including anesthesiologists (n=5), surgeons (n=2), perioperative nurses (n=4), and surgical unit nurses (n=3). Interviews with medical providers revealed widespread concerns of inadequate postoperative monitoring during recovery and after transfer to the units due to the short duration in the recovery area, with primary safety concerns being hemodynamic instability, respiratory complications, and mental status due to residual anesthetics (Figure 1). The greatest needs identified were the establishment of a designated PACU, and additional nursing staff and physiologic monitoring (Figure 2).

## Discussion

Pulse oximetry enabled the detection of hypoxemic events at HNC that were previously unrecognizable, providing valuable information regarding clinical status during the high-risk period immediately after surgery. In this population, events were typically of short duration and resolved primarily with stimulation, without any significant patient complications. However, delayed identification and lack of early intervention can lead to severe complications and preventable morbidity and mortality. Respiratory complications, commonly due to airway obstruction, hypoventilation, atelectasis, bronchospasm, and acute respiratory failure, are leading causes of increased hospital stay and healthcare costs following major surgeries [10]. Thus, the use of pulse oximetry is recommended in the postoperative period, particularly in those at an increased risk for respiratory complications [11,12]. Despite worldwide efforts to promote the expansion of pulse oximetry, there is a discrepancy between the actual and the expected use of pulse oximeters in LMIC settings, though this improved with the implementation of donated devices [6]. This report demonstrates the ongoing need for assistance in supplying portable monitors to resource-limited public hospitals in Guatemala.

In this cohort of postoperative patients who were not placed on supplemental oxygen, 10% of patients experienced a desaturation event. This is consistent with prior studies of oximetry in LMICs who have reported an incidence of EPH ranging from 4% to 24% [5,13,14]. The high rate of orthopedic and trauma surgeries and low rate of general anesthesia compared to regional techniques reflect the standard practices in this hospital. Regional anesthesia is often preferentially used in many LMIC settings for resource optimization and patient safety, where there may be lower rates of hypoxemia compared to high-income countries, where general anesthesia is more common [15]. Patients who receive general anesthesia have been reported to have an eight times greater chance of developing hypoxemia than those who receive regional anesthesia. Factors that have been shown to contribute to postoperative hypoxia include type of anesthesia, type of surgery, age, severe pain, history of obstructive sleep apnea, and duration of anesthesia [13,14]. In this patient cohort, a statistically significant association was only observed with age, though the incidence of desaturation was nearly doubled in patients who received general anesthesia. The small sample size may contribute to a lack of statistical significance observed with other factors, such as anesthesia type, in this population. Age has been associated with EPH in prior studies in LMICs [13,14]. Advanced age can result in a decline in pulmonary function and increased sensitivity to pharmacologic agents and has been shown to be an independent risk factor for postoperative pulmonary complications [16,17]. In this hospital setting with limited resources, the identification of higher-risk patients can be useful to allocate resources, such as oxygen or continuous physiologic monitoring, or modify care plans to prevent postoperative complications.

The clinical relevance of hypoxemic events can be context-dependent. Though oxygen desaturations of short duration are often tolerated in healthy patients without significant organ damage, even mild hypoxemia in critically ill patients can be an independent risk factor for increased mortality [18]. Brief episodes of hypoxemia can be associated with worse long-term outcomes and significant physiological responses such as respiratory changes, increased heart rate, and vasoconstriction, which can exacerbate cardiovascular risks [19,20]. These physiologic perturbations may also lead to cognitive impairment, which could impact patient management and resource allocation in settings such as HNC with limited patient supervision. Further analysis of the impact of hypoxemia on long-term clinical outcomes in this patient population is outside of the scope of this study but is warranted to better understand the clinical implications of EPH in resource-limited settings.

In addition to cost and device availability, human factors, such as lack of expertise and training, have been identified as barriers to implementing pulse oximetry in LMICs [21]. Over the course of the study, local staff were trained on the new devices and provided with printed manuals and instructions for use and troubleshooting. Although care was deferred to the local team and their practice was observed, there were collaborative discussions about patient management and protocols for staff related to postoperative observation and response to device alerts, in accordance with hospital policies and available resources. Ongoing education and training are essential for the sustainable integration of new technology and practices in diverse health systems [22]. Ensuring a reliable power source for device operation is also an important factor in considering the feasibility of implementation. The devices in this study are rechargeable and there are no concerns about access to a reliable power supply for recharging. Advancements in portable monitoring technology, such as pulse oximetry and capnography, have facilitated global application and use, and the ongoing promotion of these devices should be prioritized.

A significant observation of this study was the short duration in the recovery area and the lack of direct supervision and patient monitoring after surgery. The limited average time of only 14 minutes in the immediate postoperative area, with a short duration of pulse oximetry monitoring and rapid transfer to the wards, where no monitoring occurs, could contribute to a lower number of EPH and result in missed events that could occur during prolonged recovery from anesthesia. In contrast, the length of stay for PACU monitoring in a high-income setting can typically range from 1 to 5 hours [23]. Although one study in Ethiopia observed the majority of hypoxemic events in the first 10 minutes after surgery, hypoxemia still occurred during the first 30 minutes of follow-up in the PACU [24]. This concern was expressed by nearly all HNC team members, who acknowledge the lack of monitoring in the wards, where there are high patient-to-nurse staffing ratios, limited training, and a lack of equipment or oxygen supplies. These challenges highlight the importance of a designated PACU for safe postoperative recovery. The lack of a PACU has been identified in nearly 20% of public hospitals in Guatemala and has also been reported in other LMICs [7,25-27]. As expressed by the HNC providers, a designated PACU would improve safety by prolonged physiologic monitoring of higher-risk postoperative patients by trained personnel with improved staffing ratios and proximity to the operating room and anesthesia team and reduce the burden on the hospital units’ nursing staff. Timely nursing care can reduce complications including hypoxemia, hypotension, pain, and nausea, but requires adequate staffing, training, and protocols [28]. Based on the reviews of the literature, investment in increased perioperative nurse staffing and a designated PACU could potentially be a cost-effective strategy to improve care quality and patient outcomes in this resource-limited setting [29].

Limitations

A limitation of this study is the small sample size from a single center. While the sample size of 100 subjects enabled the detection of a statistically significant association with EPH and age in this cohort, the ability to detect small differences and associations with other risk factors may have been limited, especially when correcting for multiple testing. The use of convenience sampling could limit generalizability to other centers but was performed to analyze the true surgical population in this hospital setting, though temporal trends in surgical procedures and patient demographics could exist. Additionally, the brief monitoring period may have prevented additional findings of EPH, which could have been captured with prolonged postoperative monitoring. As previously discussed, this is an area of active concern that warrants further investigation.

## Conclusions

Portable pulse oximetry is an effective tool for detecting previously unrecognized hypoxemia in postoperative patients in resource-limited settings. Despite widespread efforts to expand the use of portable pulse oximetry, this report demonstrates the ongoing need for the implementation of this essential technology in resource-limited settings. The patient and system risk factors identified in this study can inform future strategies to improve postoperative patient safety in public hospitals in Guatemala. Extended patient monitoring and the establishment of a dedicated PACU with adequate staffing and equipment are necessary to avoid preventable patient complications and ensure optimal patient outcomes after surgery.

## References

[1] Merry AF, Cooper JB, Soyannwo O, Wilson IH, Eichhorn JH (2010). International Standards for a Safe Practice of Anesthesia 2010. Can J Anaesth.

[2] Peterson ME, Mattingly AS, Merrell SB, Asnake BM, Ahmed I, Weiser TG (2022). Pulse oximeter provision and training of non-physician anesthetists in Zambia: A qualitative study exploring perioperative care after training. BMC Health Serv Res.

[3] Enright A, Merry A, Walker I, Wilson I (2016). Lifebox: A global patient safety initiative. A A Case Rep.

[4] Burn SL, Chilton PJ, Gawande AA, Lilford RJ (2014). Peri-operative pulse oximetry in low-income countries: A cost-effectiveness analysis. Bull World Health Organ.

[5] Sama HD, Maman AF, Walker IA (2015). Incidence of hypoxia and related events detected by pulse oximeters provided by the Lifebox Foundation in the maternity unit at Sylvanus Olympio University Teaching Hospital, Togo. J Anesth.

[6] Walker IA, Merry AF, Wilson IH (2009). Global oximetry: An international anaesthesia quality improvement project. Anaesthesia.

[7] Zha Y, Truché P, Izquierdo E (2021). Assessment of anesthesia capacity in public surgical hospitals in Guatemala. Anesth Analg.

[8] Asturias EJ, Heinrichs G, Domek G (2016). The Center for Human Development in Guatemala: An innovative model for global population health. Adv Pediatr.

[9] von Elm E, Altman DG, Egger M, Pocock SJ, Gøtzsche PC, Vandenbroucke JP; STROBE Initiative (2007). The Strengthening the Reporting of Observational Studies in Epidemiology (STROBE) statement: Guidelines for reporting observational studies. Lancet.

[10] Ferreyra G, Long Y, Ranieri VM (2009). Respiratory complications after major surgery. Curr Opin Crit Care.

[11] Lam T, Nagappa M, Wong J, Singh M, Wong D, Chung F (2017). Continuous pulse oximetry and capnography monitoring for postoperative respiratory depression and adverse events: A systematic review and meta-analysis. Anesth Analg.

[12] American Society of Anesthesiologists Task Force on Perioperative Management of patients with obstructive sleep apnea (2014). Practice guidelines for the perioperative management of patients with obstructive sleep apnea: An updated report by the American Society of Anesthesiologists Task Force on Perioperative Management of patients with obstructive sleep apnea. Anesthesiology.

[13] Quintero-Cifuentes IF, Pérez-López D, Victoria-Cuellar DF, Satizábal-Padridín N, Billefals-Vallejo ES, Castaño-Ramírez DA, Beltrán-Osorio LD (2018). Incidence of early postanesthetic hypoxemia in the postanesthetic care unit and related factors. Colomb J Anesthesiol.

[14] Andualem AA, Yesuf KA (2022). Incidence and associated factors of postoperative hypoxemia among adult elective surgical patients at Dessie Comprehensive Specialized Hospital: An observational study. Ann Med Surg (Lond).

[15] Dohlman LE, Kwikiriza A, Ehie O (2020). Benefits and barriers to increasing regional anesthesia in resource-limited settings. Local Reg Anesth.

[16] Canet J, Gallart L, Gomar C (2010). Prediction of postoperative pulmonary complications in a population-based surgical cohort. Anesthesiology.

[17] Neto AS, da Costa LG, Hemmes SN (2018). The LAS VEGAS risk score for prediction of postoperative pulmonary complications: An observational study. Eur J Anaesthesiol.

[18] SRLF Trial Group (2018). Hypoxemia in the ICU: Prevalence, treatment, and outcome. Ann Intensive Care.

[19] Khirfan G, Naal T, Abuhalimeh B, Newman J, Heresi GA, Dweik RA, Tonelli AR (2018). Hypoxemia in patients with idiopathic or heritable pulmonary arterial hypertension. PLoS One.

[20] Hajipour M, Hirsch Allen AJ, Beaudin AE (2024). All obstructive sleep apnea events are not created equal: The relationship between event-related hypoxemia and physiologic response. Ann Am Thorac Soc.

[21] Sheikh M, Ahmad H, Ibrahim R, Nisar I, Jehan F (2023). Pulse oximetry: Why oxygen saturation is still not a part of standard pediatric guidelines in low-and-middle-income countries (LMICs). Pneumonia (Nathan).

[22] Khan IA, Karim HM (2023). Anesthesia services in low- and middle-income countries: The fragile point for safe surgery and patient safety. Cureus.

[23] Ganter MT, Blumenthal S, Dübendorfer S (2014). The length of stay in the post-anaesthesia care unit correlates with pain intensity, nausea and vomiting on arrival. Perioper Med (Lond).

[24] Wolde GD, Awol MA, Obsa MS, Wesene NG, Gemechu AD, Tadesse EN (2018). Magnitude and associated factors of immediate postoperative hypoxemia among elective surgical procedures at Tikur Anbessa Specialized Hospital, Addis Ababa, Ethiopia. J Anesth Clin Res.

[25] Shahbaz S, Zakar R, Fischer F (2021). Anesthesia health system capacities in public hospitals of Punjab, Pakistan. Inquiry.

[26] Kifle F, Belihu KD, Beljege BZ (2021). Perioperative care capacity in East Africa: Results of an Ethiopian national cross-sectional survey. IJS Global Health.

[27] Charuluxananan S, Thienthong S, Rungreungvanich M, Srirojanakul W, Punjasawadwong Y, Sriprajittichai P (2009). A survey of post anesthetic pain management in Thailand. J Med Assoc Thai.

[28] Mert S (2023). The significance of nursing care in the post-anesthesia care unit and barriers to care. Intensive Care Res.

[29] Griffiths P, Saville C, Ball J, Dall'Ora C, Meredith P, Turner L, Jones J (2023). Costs and cost-effectiveness of improved nurse staffing levels and skill mix in acute hospitals: A systematic review. Int J Nurs Stud.

